# Evaluation of the Safety and Efficacy of Teneligliptin at a Higher Dose in Indian Type 2 Diabetes Patients: A Retrospective Analysis

**DOI:** 10.7759/cureus.6812

**Published:** 2020-01-29

**Authors:** Asis Mitra, Saswati Ray

**Affiliations:** 1 Internal Medicine, Ruby General Hospital, Kolkata, IND; 2 Physiology, Jagannath Gupta Institute of Medical Science, Kolkata, IND

**Keywords:** type 2 diabetes mellitus, teneligliptin, add on therapy, metformin, glycemic parameters

## Abstract

Background and aim

While diabetes mellitus (DM) is occupying the topmost global epidemic position, India is harboring a challenging number of type 2 DM patients in the world. This devastating picture of the health sector in India requires the availability of more cost-effective, context-specific, and safer drugs for DM management. This study aimed to evaluate the safety and efficacy of teneligliptin in Indian patients with type 2 DM inadequately controlled with diet, exercise, and a maximal dose of metformin treatment.

Materials and methods

This was a retrospective, observational, and single-center study conducted at a diabetic clinic in India in type 2 DM patients who have been treated with teneligliptin 40 mg once daily as add-on therapy with diet, exercise, and the maximal tolerable dose of metformin for three months. The study was observational, where the data collection was through self-reporting and an observational study conducted over one year (September 2018 to August 2019). A total of 100 patients were enrolled in the study (male 69% and female 31%). Patients with available data for fasting plasma glucose (FPG), postprandial plasma glucose (2h PPG), glycated hemoglobin (HbA1c), renal function parameters, such as urinary albumin to creatinine ratio (UACR), and electrocardiogram (ECG) at baseline and three months after treatment were enrolled in the study.

Results

There was a signiﬁcant reduction in fasting blood sugar (P=<0.001), postprandial blood sugar (P=<0.001), and HbA1c (P=<0.001) at the end of the three months treatment in comparison to the baseline level and in the primary outcomes of this study as compared to baseline. The teneligliptin treatment did not cause any significant reduction in body mass index (BMI) before and after treatment. When we compared the secondary outcomes, the indicator of renal function as expressed through the albumin-to-creatinine ratio (ACR; P=0.052), there was a borderline change in ACR from baseline to three months. The mean corrected QT interval at screening baseline was 429.7 ± 8.89 milliseconds while after three months, it was 429.1 ± 8.68 milliseconds, which was statistically insignificant.

Conclusion

The current results demonstrated a high level of efficacy as an add-on therapy of teneligliptin at a high dose with inadequately controlled type 2 DM subjects in India. The study results also indicate the good tolerance of this drug with no critical adverse event in this study design.

## Introduction

Diabetes mellitus (DM), the global epidemic, is affecting not only developed countries but also posing a tremendous burden in developing countries [1]. It is indeed a global health challenge for the 21st century [1]. If the present trend of DM continues, by 2045, almost 134 million people will have diabetes in the world and by 2030, DM may afflict up to 79.4 million individuals in India while China (42.3 million) and the United States (30.3 million) will also see significant increases in those affected by the disease [2-3]. Based on the statistics of the International Diabetes Federation (IDF) 2015 report, around 69.2 million diabetic patients live in India, the second-most highly prevalent country after China (109.6 million) [4]. More recent statistics evidenced that approximately ~73 million people were diagnosed with DM in India in 2017 [2]. Thus, undoubtedly, India is a country with an overburden of DM [5]. According to the health projection report of IDF, as estimated in 2015, if the current trend of DM continues, by 2040, India will have about 123.5 million DM patients [4]. Among the uncountable factors behind this unexpected increase of DM prevalence in India, the one that must be stated here the rapid shift in the Indian economy [3,5].

To combat the situation, India is taking appropriate and context-specific government interventions, and combined efforts from all the stakeholders of the society are highly required and demanded. An urgent therapeutic approach, which is cost-effective, and safer drugs for its management, is highly essential [6]. To date, there are several guidelines to control and avoid diabetic complications worldwide. The very recent target to prevent and control diabetic complications is a glycated hemoglobin (HbA1c) less than 7.0% as a target, which can be achieved through a combination of diet, exercise therapy, and pharmacotherapy [7]. Thus, several pharmacological interventions of DM are available, and the individual prescription depends on the patient’s medical condition, the pharmacological properties of the drug, including its side-effect proﬁle, namely, the incidence of hypoglycemia [8].

New therapies with the least risk of hypoglycemia are now mostly used. If we inhibit the degradation of glucagon-like peptide-1, the dipeptidyl peptidase-4 (DPP-4) inhibitors promote insulin secretion and suppress glucagon secretion [9]. Due to the unique mode of action on the glucose concentration and the low risk of induction of hypoglycemia, DPP-4 inhibitors have attracted a lot of research interests from basic science to clinical trials from pharmacokinetics to pharmacodynamics, including safety and efficacy [10-11]. The independent mechanisms of action of these drugs and the low risk of hypoglycemia provide support for combination therapy as a therapeutic option. Indeed, prior studies have demonstrated that combination therapy with an SGLT2 inhibitor and a DPP-4 inhibitor was effective and well-tolerated [12-14]. Among various DPP-4 inhibitors, teneligliptin has been approved for the management of type 2 DM in some countries, namely, Japan (2012), South Korea (2014), and India (2015) [3]. In Japan, the drug teneligliptin has been licensed for administration at standard (20 mg/day) and high (40 mg/day) doses for glycemic control [15]. Teneligliptin has shown good tolerance and is capable of glycemic control as monotherapy or add-on therapy in patients with type 2 DM [3].

Controversial evidence exists on the effects of teneligliptin on lowering the HbA1c level in type 2 DM patients. A previous study showed a statistically non-significant difference in results on HbA1c levels mediated by the standard dose (−0.9% vs. placebo) and a high dose of teneligliptin (−1.0% vs. placebo) [16]. A high dose of teneligliptin exerts proper glycemic control in DM patients [17]; it also takes part in the improvement of insulin resistance [18-19].

The current study aims to further add the evidence that teneligliptin at a dose of 40 mg/day is even effective on the related parameters of glycemic control in type 2 DM patients who are already on a diet, exercise program, and the maximally tolerated dose of metformin therapy. We believe the present study will add some pieces of evidence on the oral use of teneligliptin at a high dose with type 2 DM patients' HbA1c ranging from 7-9%. Here, we must state that the study subjects in this analysis had the inadequate control status of DM although they were on metformin monotherapy with lifestyle interventions (diet and exercise program).

## Materials and methods

Study population

This was a retrospective, observational, and single-center study conducted at a diabetic clinic in India on type 2 DM patients who were on treatment with teneligliptin 40 mg once daily as add-on therapy with diet, exercise, and the maximally tolerable dose of metformin for three months. The study was observational, where data collection was through self-reporting and the observational approach over one year (September 2018 to August 2019). A total of 100 patients were included in the current retrospective analysis (male 69% and female 31%). All the ethical issues and approvals were processed in terms of both local and international guidelines before the study started. The trial was conducted by the Declaration of Helsinki and Good Clinical Practice (GCP).

Inclusion criteria

In this study, the enrolled type 2 DM patients had an HbA1c level of >7% and less than <9%. Type 2 DM patients inadequately controlled with diet, exercise, and the maximum tolerable dose of metformin were included in the study.

Exclusion criteria

We excluded the following study subjects in the current study: those with a history of pancreatitis, pregnant patients, type 1 DM patients, and patients with chronic liver diseases or a history of cardiac arrhythmia.

Data used

There was no randomization in the present study. Patients with available data for fasting plasma glucose (FPG), two-hours postprandial plasma glucose (2h PPG), HbA1c, renal function parameters, such as urinary albumin to creatinine ratio (UACR), and electrocardiogram (ECG) at baseline and three months post-treatment were enrolled in the study. Also, we had general information about age, sex, body weight, and BMI. The QT interval in ECGs is a measure of cardiac repolarization, and any drug that causes an increase in the QT interval may enhance the risk of cardiovascular (CV) events, and, hence, the QT interval was focused on ECG in the present study. We strictly maintained the privacy and confidentiality of data throughout the study period. We also monitored and reported on the patients on all adverse reactions. Efficacy and safety assessments were performed at 12 weeks and were compared with those at baseline levels with a statistical tool.

Here, baseline data were the measurements done before the initiation of teneligliptin treatment. The efficacy outcome was classified as primary and secondary and considered the change in each study parameter after three months of teneligliptin treatment, namely, (a) HbA1c, (b) fasting blood sugar, (c) two-hour post-prandial blood sugar, (d) albumin to creatinine ratio, (e) corrected QT interval (QTc), and (f) BMI. The QT interval needs to be corrected for heart rate, which enables a comparison with reference values. As heart rate is a crucial indicator of repolarization length, many correction formulae have been developed to calculate a QTc value corresponding to a QT value normalized at a heart rate of 60 beats/minute.

Statistical analysis

Statistical analysis was done with the SPSS software package (version 17, SPSS Inc., Chicago, IL, USA). All characteristics were summarized descriptively. For continuous variables, data were represented using means ± SD. For categorical data, the number and percentage were used in the data summaries. The difference between the means of analysis variables between the two independent groups was tested by an unpaired t-test. Also, an age-adjusted comparison was made by a repeated measure, analysis of covariance (ANCOVA).

## Results

In the present study, there was no missing data of enrolled patients. A total of 100 patients were enrolled for data analysis to evaluate the effect and safety issues in the current study. The mean body weight was (Mean ± SD, kg): all subjects: 71.23 ± 8.12, only male: 75.32 ± 5.70, only female: 62.13 ± 4.44.

The mean baseline BMI was 27.16 ± 1.89. From the more stratified analysis, among the enrolled patients, 15% were with the normal range of BMI, followed by overweight (75%) and obesity (10%) (Table 1). Around 96% of patients had baseline postprandial glucose levels of more than 200 mg/dl, while the moderate elevation of HbA1c level was seen in 88% enrolled study subjects [20]. Around 80% of study subjects had normal kidney function, as demonstrated by normal ACR. Approximately 65% of patients had a normal, baseline-corrected Q-T interval [21].

**Table 1:** Baseline demographic and clinical characteristics of all patients ACR: albumin to creatinine ratio

Patients characteristics	Number of patients, n (%)
Total number of patients	100 (100%)
Gender	
Male	69 (69%)
Female	31 (31%)
Baseline body mass index (BMI)	
Normal weight (BMI 18.5–24.9 kg/m^2^)	15(15%)
Overweight (BMI 25–29.9 kg/m^2^)	75(75%)
Obese (BMI ≥30.0 kg/m^2^)	10 (10%)
Baseline postprandial blood glucose level (mg/dL)	
≤200	4 (4%)
>200	96 (96%)
Hemoglobin A1c (HbA1c, %)	
Mild elevation (6%–7.99%)	12 (12%)
Moderate elevation (8%–9.99%)	88 (88%)
Baseline albumin-to-creatinine ratio (ACR)	
Normal (ACR<30 mg/g)	79 (79%)
Microalbuminuria (ACR 30-300 mg/g)	21 (21%)
Baseline corrected Q-T interval (QTc)	
Normal (Male<430 ms & Female<450 ms)	65 (65%)
Borderline (Male 431-450 ms & Female 451-470 ms)	35 (35%)

There was a signiﬁcant reduction in fasting blood sugar (P=<0.001), postprandial blood sugar (P=<0.001), and HbA1c (P=<0.001) at the end of the three months treatment in comparison to baseline level (Table 2), the primary outcomes of this study. As compared to baseline, the average decrease in fasting blood sugar was -42.7 ± 1.01 mg/dL (28.9%), that of postprandial blood glucose was -49.8 ± 2.56 mg/dL (21.8%), and that of HbA1c was -0.98 ± 0.01% (11.8%) (Table 2). These results indicate the significant improvement in glycemic control before and after teneligliptin treatment.

**Table 2:** Effect of teneligliptin added to standard treatment on FBG, PPBG, and HbA1c Note: *Significant at the 5% level of significance (P<0.05)

Parameters	Baseline	3 months	T-value	P-value	Mean change
	Mean ± SD	Mean ± SD			
Fasting blood glucose (FBG, mg/dL)	147.8 ± 8.19	105.1 ± 7.18	39.18	<0.001*	-42.7 ± 1.01
Postprandial blood glucose (PPGB, mg/dL)	229.7 ± 15.76	179.9 ± 13.20	24.18	<0.001*	-49.8 ± 2.56
Postprandial blood glucose (mg/dL) ≤200	199.5 ±1.0	178.7 ± 12.3	3.36	0.001*	-20.8 ± 11.3
Postprandial blood glucose (mg/dL) >200	230.9 ±14.8	203.6 ± 2.19	4.11	<0.001*	-27.3 ± 12.6
Hemoglobin A1c (HbA1c, %)	8.22 ± 0.20	7.24 ± 0.19	34.45	<0.001*	-0.98 ± 0.01
HbA1c-Mild elevation (6%–7.99%)	7.88 ± 0.04	7.24 ± 0.20	11.17	<0.001*	-0.64 ± 0.16
HbA1c-Moderate elevation (8%–9.99%)	8.26 ± 0.17	Absent			

The teneligliptin treatment did not cause any significant reduction in BMI before and after treatment (Table 3) as a secondary outcome considered in the current study. When we compared the secondary end-points, the indicator of renal function as expressed through the albumin-to-creatinine ratio (ACR), there was a borderline change in ACR from baseline to three months (P=0.052) (Table 3). The mean QT interval at the screening at baseline was 429.7 ± 8.89 milliseconds while after three months, it was 429.1 ± 8.68 milliseconds, which was statistically insignificant (P=0.653) (Table 3).

**Table 3:** Effect of teneligliptin added to standard treatment on secondary outcomes Note: *Significant at 5% level of significance (P<0.05), screening vs. end of the study

Parameters	Baseline	3 months	T-value	P-value
	Mean ± SD	Mean ± SD		
Body mass index (BMI, kg/m^2^)	27.16 ± 1.89	26.96 ± 1.85	0.74	0.461
Normal weight (BMI 18.5–24.9 kg/m^2^)	24.45 ± 0.61	24.40 ± 0.57	0.23	0.822
Overweight (BMI 25–29.9 kg/m^2^)	27.26 ±1.41	27.33 ±1.39	-0.31	0.754
Obese (BMI ≥30.0 kg/m^2^)	30.22 ± 0.33	30.20 ± 0.40	0.11	0.914
Albumin-to-creatinine ratio (ACR, mg/g)	26.75 ± 20.66	21.69 ± 15.64	1.95	0.052*
Normal (ACR<30 mg/g)	19.27 ± 6.00	17.06 ± 6.24	2.31	0.022*
Microalbuminuria (ACR 30-300 mg/g)	54.90± 30.33	50.14± 24.40	0.49	0.627
Corrected Q-T interval (QTc, ms)	429.7 ± 8.89	429.1 ± 8.68	0.45	0.653
QTc-Normal (Male<430 ms & Female<450 ms)	425.9 ± 8.56	425.7 ± 8.45	0.83	0.906
QTc-Borderline (Male 431-450 ms & Female 451-470 ms)	436.8± 3.41	436.2 ± 3.17	0.85	0.400

From the retrospective database, there was no incidence of hypoglycemia or any other reported adverse effect in any of the patients, implying the well-tolerance of all enrolled patients to the drug.

Later, we also made the age-adjustment comparison in concern to all study outcome parameters, either primary or secondary (Table 4). The results were consistent for all glycemic parameters as that of Table 2.

**Table 4:** Effect of teneligliptin added to standard treatment on study outcomes by age adjustment Note: *Significant at 5% level of significance (P<0.05)

Parameters	Baseline	3 months	F	P-value
	Mean ± SD	Mean ± SD		
Body mass index (BMI, kg/m^2^)	27.13 ± 1.90	26.98 ± 1.86	16.60	<0.001*
Fasting blood glucose (FBG, mg/dL)	147.8 ± 8.19	105.1 ± 7.18	186.69	<0.001*
Postprandial blood glucose (PPBG, mg/dL)	229.7 ± 15.76	179.9 ± 13.20	39.77	<0.001*
Hemoglobin A1c (HbA1c) (%)	8.22 ± 0.20	7.24 ± 0.19	238.74	<0.001*
Albumin-to-creatinine ratio (ACR, mg/g)	26.75 ± 20.66	21.69 ± 15.64	0.128	0.721
Corrected Q-T interval (QTC, ms)	429.7 ± 8.89	429.1 ± 8.68	0.06	0.810

No gender-based vast difference was observed in an age-adjusted analysis in terms of glycemic parameters (Table 5). Also, there was no significant change in gender-based (ACR) albumin-to-creatinine ratio (P=0.882) and (P=0.150) (Table 5). There was no signiﬁcant change in the gender-based analysis in the QT interval (P=0.641) and QTc interval (P=0.787) at baseline and the end of the study (Table 5).

**Table 5:** Effects of teneligliptin added to standard treatment by age adjustment (gender-based) Data shown: Mean ± SD, *Significant at 5% level of significance (P<0.05)

	Male				Female			
	Baseline	3 months	F	P-value	Baseline	3 months	F	P-value
Body mass index (BMI, kg/m^2^)	28.07 ± 1.42	27.89 ± 1.38	12.80	0.001*	25.06 ± 0.97	24.95 ± 0.98	5.39	0.027*
Fasting blood glucose (FBG, mg/dL)	149.0 ± 8.26	106.1 ± 7.39	111.74	<0.001*	145.2 ± 7.51	102.9 ± 6.23	77.32	<0.001*
Postprandial blood glucose (PPBG, mg/dL)	229.0 ± 14.97	179.5 ± 12.74	26.16	<0.001*	231.2 ± 17.55	180.9 ± 14.36	11.93	0.002*
Hemoglobin A1c (HbA1c) (%)	8.22 ± 0.20	7.25 ± 0.19	138.99	<0.001*	8.21 ± 0.22	7.22 ± 0.22	95.75	<0.001*
Albumin-to-creatinine ratio (ACR, mg/g)	28.12 ± 22.73	22.80 ± 16.21	0.02	0.882	23.71 ± 14.97	19.23 ± 14.24	2.18	0.150
Corrected Q-T interval (QTc, ms)	429.3 ± 9.25	428.6 ± 8.97	0.22	0.641	430.7 ± 8.09	430.4 ± 7.97	0.07	0.787

Finally, regarding BMI after age adjustment, a significant change was observed in total participants (Table 4) and in the gender-based analysis in Table 5 from baseline to the end of treatment.

**Figure 1:**
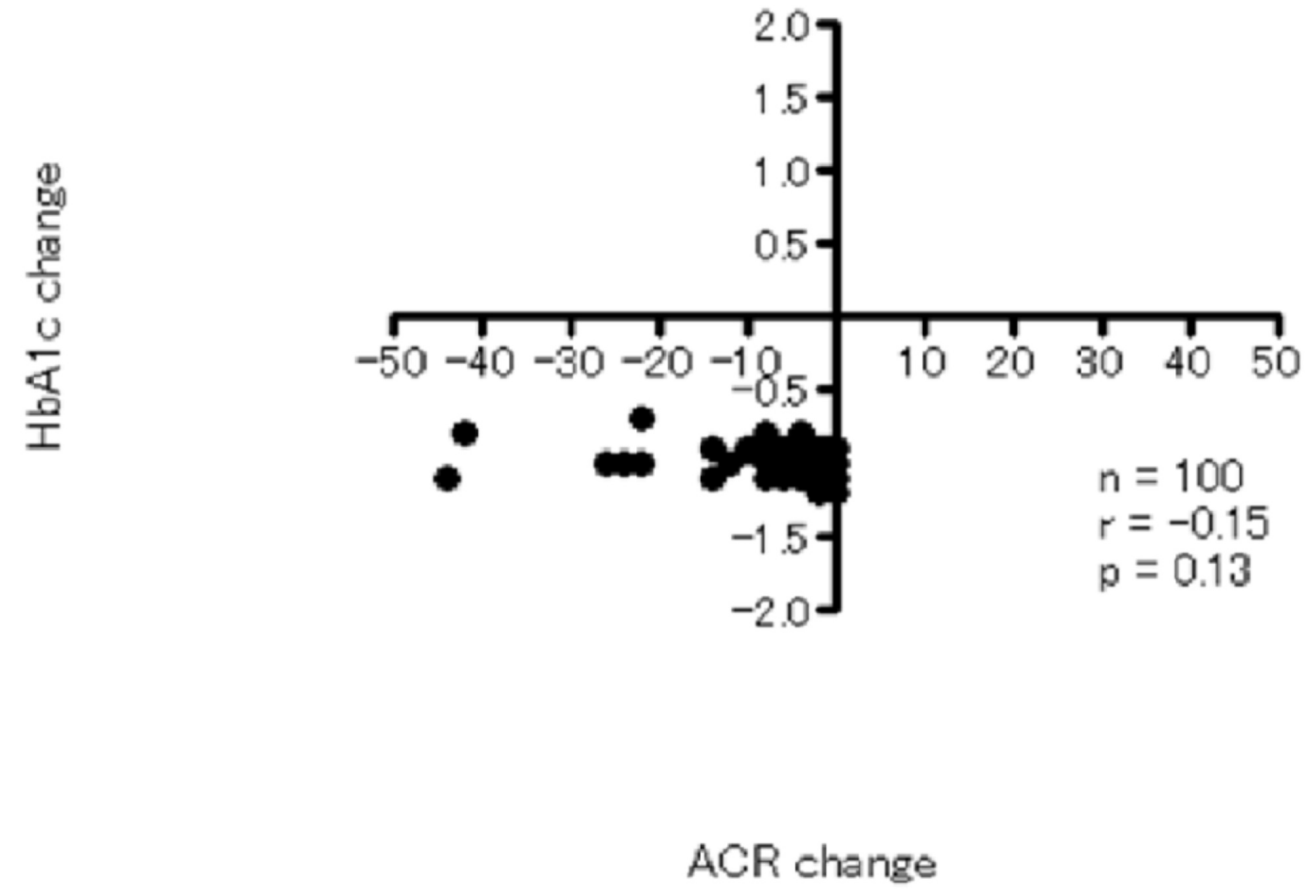
Correlation analysis between change of three months-baseline of HbA1c and ACR HbA1c: glycated hemoglobin; ACR: albumin to creatinine ratio

We also made a correlation analysis on a change of after treatment vs. baseline for HbA1c and ACR in total participants but no significant correlation was observed (Figure 1).

## Discussion

The findings of the present study, through a retrospective analysis, demonstrate that teneligliptin was effective in a significant reduction on the glycemic parameters at a comparatively high dose (40 mg oral, daily) as an add-on therapy for inadequately controlled type 2 DM patients who are already on regimen of diet, exercise, and the maximal tolerable dose of metformin. Kidney and cardiac functions were unaffected while this treatment regimen exerted differential effects on BMI in a condition with or without age adjustment. There was no gender-specific difference in glycemic outcomes in the present study.

The present study analyzed the essential parameters of glycemic control, namely, fasting blood glucose (FBG), 2hPPG, and HbA1c both at baseline and three months after teneligliptin treatment with or without age adjustment (Table 2 and Table 4). The effectiveness of teneligliptin in reducing glycemic burden as an add-on to monotherapy has been shown in various studies. A recent study conducted in India demonstrated patients with type 2 DM who had inadequate control of glycemic parameters with monotherapy, teneligliptin (20 mg/day) as add-on to existing treatment significantly reduced HbA1c, FPG, and PPG at 12 weeks, the same duration as that of current study implying again the potential efficacy of addition of teneligliptin on existing monotherapy on uncontrolled glycemic states of type 2 DM patients [22]. In another 16 weeks trial where teneligliptin was added at 20 mg/day to metformin showed a significant reduction in HbA1c (adjusted mean difference -0.90% vs. -0.12%, p<0.0001) and FPG (adjusted mean difference -1.10 mmol/L vs 0.15 mmol/L, p<0.0001) in type 2 DM patients as compared to the placebo group [23]. Another study conducted in Korean type 2 DM patients with combination therapy of metformin and teneligliptin showed comparable results. Similar findings as of the current research were demonstrated through a substantial reduction of HbA1c and FPG with the addition of teneligliptin to glimepiride monotherapy in type 2 DM Japanese patients [24]. In a clinical setting, baseline HbA1c is a significant predictor of glycemic response [25]. Collectively, the presented several pieces of evidence from different geographic regions suggests that teneligliptin as an add-on treatment to monotherapy is effective in lowering glycemic parameters levels irrespective of baseline antidiabetic medication. Also, the age-adjustment did not affect the findings on glycemic parameters in the current study design (Table 4).

As a secondary outcome, we calculated BMI in the current study, and we did not find any significant effects of the drug combination therapy used in the present study in an age-unadjusted analysis (Table 3) in total participants. But in age-adjusted analysis, there was a significant reduction in BMI before and after teneligliptin addition. Inconsistent results have been observed in past studies, although most of the studies considered the impact of DPP-4 inhibitors on the body as neutral [26]. In another study, combination therapy of metformin with teneligliptin did not show any significant change in body weight between the recording time baseline vs. 24 weeks of treatment duration [27]. Thus, based on current findings, we conclude that teneligliptin as an additional treatment to already existing monotherapy did not exert any detrimental effect on BMI in type 2 DM patients.

As type 2 DM is a potential risk factor for cardiovascular disease (CVD) and chronic kidney disease (CKD), the present study also investigated the impact of teneligliptin as an add-on therapy to metformin. There was a marginal improvement of kidney function, as demonstrated by the urinary ACR level in comparison to the baseline level in the total analysis (Table 3). In the stratified analysis, a statistically significant effect was only seen with patients of normal ACR (Table 3). Teneligliptin contributes to reno-protection, as evidenced by the reduction of albuminuria in type 2 DM patients [28]. Teneligliptin switching from sitagliptin improves endothelial function and reduces renal and vascular oxidative stress in patients with type 2 DM and CKD compared with those taking sitagliptin [28]. The effect of teneligliptin was independent of improvement in glucose control, and also, the amount of albuminuria exhibited no significant alteration between teneligliptin and sitagliptin treatment [28]. Thus, the present findings were consisted of past research in terms of reno-protection exerted by added on therapy of teneligliptin in type 2 DM patients.

In the present study also, there was no significant change in cardiac function as measured by QTc (Table 3) with combination therapy of teneligliptin and metformin in type 2 DM patients. In the stratified analysis also, there was no change in QTc at baseline, and after 12 weeks of treatment (Table 3). The current findings on QTc were similar to a past study where no QT prolongation was detected with 40 mg/day of teneligliptin, which is the maximal dosage used in clinical practice, in a study conducted in type 2 DM patients in India [29]. Indeed, some non-antiarrhythmic drugs exert undesirable effects by delaying cardiac repolarization, a result which is determined as the prolongation of the QT interval on the surface ECG [29]. Thus, the current findings add to the evidence on the non-impairment of cardiac function in type 2 DM in combination therapy with teneligliptin even at a high dose.

In the current study, teneligliptin was used as an add-on therapy to another antidiabetic agent metformin (the maximally tolerated dose for each patient), and this combination therapy showed good tolerance to our study participants. The current study subjects were also on lifestyle interventions such as diet and exercise. Either through self-reporting or monitoring by healthcare staff, there was no event of hypoglycemia and other known adverse effects in this retrospective analysis.

The current study used a combination approach with metformin, an insulin-sensitizing biguanide used to treat type 2 DM. This drug reduces blood glucose through the suppression of hepatic gluconeogenesis and increased insulin-stimulated glucose uptake in skeletal muscle and adipocytes [30]. A similar study design was also evident in another clinical investigation [27]. This study by Riyaz et al. also assessed the effectiveness and potential of metformin in combination with teneligliptin in Indian type 2 DM patients who did not have proper glycemic control with metformin monotherapy [27]. Here, the duration of treatment was different, 24 weeks. In this study, the primary endpoint was set at the changes in HbA1c levels from baseline to week 24, and there was a significant reduction of HbA1c from baseline to the end of the study period with good tolerance [27].

There are several limitations to the current study. There was no placebo group found from the retrospective database. Also, the dose information was absent from metformin therapy and the exercise habit. The sample size was small, only one dose (40 mg/day) was used, and an only one-time point was investigated in the current analysis.

## Conclusions

The present findings conclude that the addition of teneligliptin at a relatively high dose (40 mg/day) to metformin treatment is useful and effective for glycemic control and well-tolerated in Indian patients with type 2 DM.
